# *Arabidopsis* Histone Methyltransferase SUVH5 Is a Positive Regulator of Light-Mediated Seed Germination

**DOI:** 10.3389/fpls.2019.00841

**Published:** 2019-06-27

**Authors:** Dachuan Gu, Rujun Ji, Chunmei He, Tao Peng, Mingyong Zhang, Jun Duan, Changyun Xiong, Xuncheng Liu

**Affiliations:** ^1^Key Laboratory of South China Agricultural Plant Molecular Analysis and Genetic Improvement, Guangdong Provincial Key Laboratory of Applied Botany, South China Botanical Garden, Chinese Academy of Sciences, Guangzhou, China; ^2^Core Botanical Garden, Chinese Academy of Sciences, Guangzhou, China; ^3^College of Tropical Crops, Yunnan Agricultural University, Pu’er, China

**Keywords:** histone methylation, SUVH5, histone methyltransferase, seed germination, *Arabidopsis*

## Abstract

Plant lifecycle starts from seed germination, which is regulated by various environmental cues and endogenous hormones. Light promotes seed germination mainly by phytochrome B (PHYB) during the initial phase of imbibition, which involves genome-wide light-responsive transcription changes. Recent studies indicated an involvement of multiple epigenetic factors in the control of seed germination. However, few studies have been reported about the role of a histone methyltransferase in light-mediated seed germination process. Here, we identified SUVH5, a histone H3 lysine 9 methyltransferase, as a positive regulator in light-mediated seed germination in *Arabidopsis*. Loss of function of *SUVH5* leads to decreased PHYB-dependent seed germination. RNA-sequencing analysis displayed that SUVH5 regulates 24.6% of light-responsive transcriptome in imbibed seeds, which mainly related to hormonal signaling pathways and developmental processes. Furthermore, SUVH5 represses the transcription of ABA biosynthesis and signal transduction-related genes, as well as a family of *DELAY OF GERMINATION* (*DOG*) genes via dimethylation of histone H3 at lysine 9 (H3K9me2) in imbibed seeds. Taken together, our findings revealed that SUVH5 is a novel positive regulator of light-mediated seed germination in *Arabidopsis*.

## Introduction

Plant life cycle initiates from seed germination, which is of both economic and ecologic importance (Rajjou et al., 2012). *Arabidopsis* seeds consist of embryo, single cell endosperm and testa from inside to outside (Finch-Savage and Leubner-Metzger, 2006). *Arabidopsis* seed germination includes two-step process, which is testa rupture followed by endosperm rupture (Yamaguchi, 2008; Weitbrecht et al., 2011). It is well known that seed germination is regulated by endogenous and exogenous factors, such as light, temperature, moisture, oxygen, nutrients, and multiple plant hormones (Finch-Savage and Leubner-Metzger, 2006; De Wit et al., 2016; Shu et al., 2016).

Light is a key environmental factor in the control of seed germination. Plants perceive different parts of the light spectrum by distinct sets of photoreceptors, such as phytochromes, cryptochromes, phototropins, ZEITLUPE family, and UVR8 (Briggs and Christie, 2002; Chaves et al., 2011; Rizzini et al., 2011; Wang and Wang, 2015). Phytochromes are red and far-red light photoreceptors that play critical role in regulating seed germination in various plants species (Borthwick et al., 1952; Shinomura et al., 1994, 1996). Dark-imbibed lettuce seeds (*Lactuca sativa* L.) irradiated with red light (R) will induce germination, whereas subsequent exposure to far-red light (FR) can reverse this process (Borthwick et al., 1952). The light signaling mechanism relies on conformational conversion between inactive state (Pr) and active state (Pfr), which are photo-convertible isoforms of phytochromes (De Wit et al., 2016). In *Arabidopsis*, there are five phytochromes, designated phytochrome A (PHYA) to phytochrome E (PHYE) (Jiao et al., 2007). PHYB plays a fundamental role in the promotion of seed germination during the initiate phase of seed imbibition (Shinomura et al., 1994; Seo et al., 2008).

It’s well known that plant hormone abscisic acid (ABA) plays a predominate role in the repression of seed germination (Seo et al., 2008; Shu et al., 2016). Endogenous ABA levels are regulated by a balance of between its biosynthesis and catabolism. The major ABA biosynthesis pathway is regulated by multiple factors, including the rate limiting enzymes 9-cis-epoxycarotenoid dioxygenases (NCEDs), zeaxanthin epoxidase (ZEP) ABA1, cytosolic short-chain dehydrogenase (SDR) ABA2, molybdenumcofactor sulfurase ABA3, as well as aldehyde oxidase AAO3 (Seo and Koshiba, 2002). Seeds of triple mutant *nced5 nced6 nced9* germinate faster than the wild-type (Frey et al., 2012), whereas transgenic plants constitutively expressing *NCED6* increase ABA levels and prevent seed germination (Martinez-Andujar et al., 2011). The core ABA signaling network is composed of PYR/PYL/RCAR receptors, PP2C phosphatases, SnRK2 kinases, bZIP-type transcription factors known as ABA-responsive element (ABRE) binding factors (Raghavendra et al., 2010; Antoni et al., 2011; Hauser et al., 2011). ABI3 (ABA insensitive 3) and ABI5, two key transcription factors in ABA signal transduction, have been reported to play crucial roles in maintaining seed dormancy, and repressing seed germination (Koornneef et al., 2002; Piskurewicz et al., 2008; Kanai et al., 2010). Moreover, seed dormancy can prevent germination when environmental conditions are suitable for germination. *DELAY OF GERMINATION 1* (*DOG1*) is a master regulator in control of seed dormancy, which belongs to a plant-specific gene family with other four additional members (Bentsink et al., 2006). Loss of function of *DOG1* in *Arabidopsis* results in abolished seed dormancy and fast germination even under unfavorable conditions (Nakabayashi et al., 2012; Graeber et al., 2014).

Previous studies displayed that multiple epigenetic factors, including chromatin-remodeling factors, histone deacetylases, histone demethylases and histone methyltransferases, play diverse roles in the regulation of seed germination and dormancy (Dean Rider et al., 2003; Perruc et al., 2007; Saez et al., 2008; Cho et al., 2012; Luo et al., 2012; Zheng et al., 2012; Zhou et al., 2013; Lee et al., 2014; Liu et al., 2014; Zhao et al., 2015; Gu et al., 2017). The chromatin-remodeling factor PICKLE selectively regulates a number of genes to repress embryonic identity during germination (Dean Rider et al., 2003; Perruc et al., 2007). SWI3B, an *Arabidopsis* homolog of the yeast SWI3 subunit of SWI/SNF chromatin-remodeling complexes, plays a negative role in ABA-repressed seed germination (Saez et al., 2008). Loss of function of histone deacetylases *HDA6* and *HD2C* result in increased sensitivity to ABA and NaCl stresses during germination (Luo et al., 2012). Moreover, a recent study demonstrated histone deacetylase HDA15 interacts with Phytochrome Interacting Factor 1 (PIF1), a key negative transcription factor in light signaling pathway, in repressing light-mediated seed germination. HDA15 and PIF1 co-repress the genes associate with multiple hormonal signaling pathways and cellular processes by decreasing the histone H3 acetylation levels in the dark conditions (Gu et al., 2017). Furthermore, two histone arginine demethylases, JMJ20 and JMJ22, were found to be positive regulators in PHYB-dependent seed germination (Cho et al., 2012). JMJ20/JMJ22 increase gibberellic acid (GA) levels via removal of histone arginine methylations of GA biosynthesis genes, *GA3ox1/GA3ox2*, and ultimately promote seed germination (Cho et al., 2012). Whereas, another two histone demethylases (LDL1/LDL2) have been reported that function redundantly in repressing seed dormancy (Zhao et al., 2015). Furthermore, histone methyltransferase KYP/SUVH4 also controlled *Arabidopsis* primary seed dormancy, while another methyltransferase EFS inhibited seed germination (Zheng et al., 2012; Lee et al., 2014). However, few studies have been reported the function of a histone methyltransferase in light-mediated seed germination process.

SUVH5, a histone H3 lysine 9 methyltransferase, belonging to the SUV(R) group of SET domain proteins, has been reported to maintain transposon elements and inverted repeats silencing via histone H3K9 dimethylation (Ebbs and Bender, 2006; Rajakumara et al., 2011; Yu et al., 2017). In the present study, we identified SUVH5 as a novel component of light-mediated transcriptional regulatory network in seed germination. SUVH5 represses the expression of key seed germination-related genes, such as ABA biosynthesis and signal transduction-related genes, as well as a group of *DOG* genes by H3K9 dimethylation in imbibed seeds.

## Materials and Methods

### Plant Materials

All *Arabidopsis* plants used in this study are in Col-0 background. The *suvh5-2* (SALK_074957) and *suvh4/5/6* mutant was a kind gift from Professor Judith Bender at the University of Brown. *suvh5-2* allele was backcrossed to Col-0 for three times. *suvh4* mutant *kyp-6* (SALK_041474) was obtained from the *Arabidopsis* Information Resource Center^1^. The seeds used for germination comparison were harvested in the same batch of plants grown at 22°C under long days (16 h WL/8 h dark). Following seeds harvesting, seeds were kept in an incubator at 22°C for about 1 month to break dormancy prior to germination assays.

### Germination Assays

The PHYB-dependent seed germination assays were performed as described previously (Oh et al., 2007). Briefly, seeds were surface-sterilized and plated on half-strength Murashige-Skoog (Sigma-Aldrich) agar plates containing 0.3% sucrose and 1% phytoagar (pH 5.7). The plates were placed in an illuminated incubator with white light (80 μmol⋅m^–2^⋅s^–1^) at 22°C. 1 h after imbibition and sterilization, seeds were irradiated with far-red light (3.82 μmol⋅m^–2^⋅s^–1^) for 5 min (indicated as FR or dark conditions), or exposure to far-red light (3.82 μmol⋅m^–2^⋅s^–1^) for 5 min following irradiation with red light (13.12 μmol⋅m^–2^⋅s^–1^) for 5 min (referred as R or light conditions). The seeds were kept in the dark to calculate the germination rates at the indicated time. At least 60 seeds were used for each experimental point, and seeds harvested from three independent batches were performed for statistical analysis.

### RNA Isolation and qRT-PCR (Quantitative RT-PCR) Analysis

After FR or R treatment, the seeds were incubated in the dark at 22°C for the indicated time. The imbibed seeds were ground to powder in liquid nitrogen and total RNA was extracted with TRIZOL Reagent (Invitrogen) according to the manufacture’s protocol. After DNase I treatment, the first strand cDNA was synthesized using 2 μg total RNA according to the manufacturer’s instruction of TransScript One-Step gDNA Removal and cDNA Synthesis Super Mix Kit (TransGen, Beijing). Quantitative RT-PCR was performed by using SYBR Green Mix (Bio-Rad) in an ABI7500 Real-Time PCR System (Applied Biosystems). Three biological replicates were performed, and three technical repeats were carried out for each biological replicate. *PP2A* was used as an internal control (Czechowski et al., 2005). The primer pairs for quantitative RT-PCR are listed in Supplementary Table S4.

### RNA-Seq (mRNA Deep Sequencing) Analysis

For whole genomic transcriptome analysis, the seeds after R light treatment were incubated in the dark at 22°C for 24 h prior to RNA extraction. Total RNA was extracted as described above and an mRNA-seq library was prepared by using an mRNA Seq Kit (Illumina). RNA-seq were performed by Genepioneer Biotechnologies (Nanjing, China) with triplicate biological samples. High-quality clean reads were obtained by removing the adaptor sequences, ambiguous reads (“N” > 10%), and low-quality reads (i.e., more than 50% of bases in a read had a quality value Q ≤ 5). Then the clean reads were mapped to *Arabidopsis* genome TAIR10 using HISAT2 software with default parameters (Pertea et al., 2016). Cuffdiff^2^ was applied to detect differentially expressed genes (DEGs). Genes with more than 1.5-fold changes with statistically significance (adjusted *P*-value < 0.05) were selected. GO (gene ontology) analyses of DEGs were performed with Metascape software^3^ with a cutoff of *P* ¡ 0.05 and a minimum overlap of 3. The regulated trends of DEGs were visualized by use of heat-map made by HemI (version 1.0.1) (Deng et al., 2014). Hierarchical clustering analysis was done with the average linkage method using the HemI software. These raw sequencing data sets were deposited in NCBI-SRA database (BioProject accession number: PRJNA489162).

### ChIP-qPCR (Chromatin Immunoprecipitation and qPCR) Assays

Equal amount of Col-0 and *suvh5-2* mutant seeds were treated with R light pulse and subsequently incubated in dark at 22°C for 24 h before ChIP-qPCR analyses. ChIP-qPCR assays were performed as previously described (Gendrel et al., 2005). After fixation with formaldehyde, the chromatin was extracted and then sheared to an average length of 500 bp by sonication. The chromatin was immunoprecipitated with anti-di-methylated histone H3K9 (catalog no. 39753; Active Motif). After cross-linking reversed, the amount of each precipitated DNA fragment was detected by quantitative PCR using specific primers listed in Supplementary Table S4. The amounts of DNA after ChIP were quantified and normalized to *TA3*, the relative enrichment refers to the H3K9me2 enrichment vs. the histone H3 occupancy. Three biological replicates were performed, and three technical repeats were carried out for each biological replicate.

### Endogenous ABA Measurements

After R treatment, the seeds were incubated in the dark at 22°C for 24 h. Samples were harvested and extracted for ABA as described previously (Wu et al., 2007). Finely powdered sample (30 mg, fresh weight) was extracted with 3 mL ethyl acetate by vortexing for 30 s followed by ultrasonic extraction in ice-cold water for 20 min. Before ultrasonic extraction, 1 ng [^2^H_6_] ABA was added to the mixture as an internal standard. After centrifuging at 10000 × *g* for 5 min at 4°C, 2.9 mL supernatants were collected, and then dried under a stream of nitrogen. The residue was re-dissolved in 100 μL methanol. The supernatants were filtered through a 0.22 μm membrane, and subjected to an ultra–performance liquid chromatography/quadrupole time–of–flight mass spectrometry (UPLC–QTOF–MS) (Acquity UPLC I-Class/ Xevo^®^ G2-XS QTOF, Waters Corporation, MA, United States). Each sample (5 μL) was injected onto a Waters ACQUITY UPLC HSS T3 C18 column (2.1 mm × 100 mm, 1.8 μm). Solvent A was Milli-Q water with 0.1% (v/v) formic acid. Solvent B was acetonitrile with 0.1% (v/v) formic acid. The solvent gradient was started at 20% B, then linearly increased to 35% within 10 min, later increased to 95% B in 0.1 min and kept for 3 min. In that moment, it suddenly dropped to 20% in 0.1 min and maintain for 3 min. The flow rate was 0.4 mL/min. The column temperature was 30°C. The electrospray ionization operated on negative mode. The MS conditions were capillary voltage: 1.5 kV; source temperature: 100°C; desolvation temperature: 300°C; cone gas flow: 50 L/h; and desolvation gas flow: 600 L/h. The quantitative analysis of ABA was based on calibration curve, which was constructed by plotting the concentration of ABA standard against the peak area of [^2^H_6_] ABA. The ABA content was determined three times for each sample. Three biological replicates were performed.

## Results

### SUVH5 Is a Positive Regulator of PHYB-Dependent Seed Germination

**FIGURE 1:**
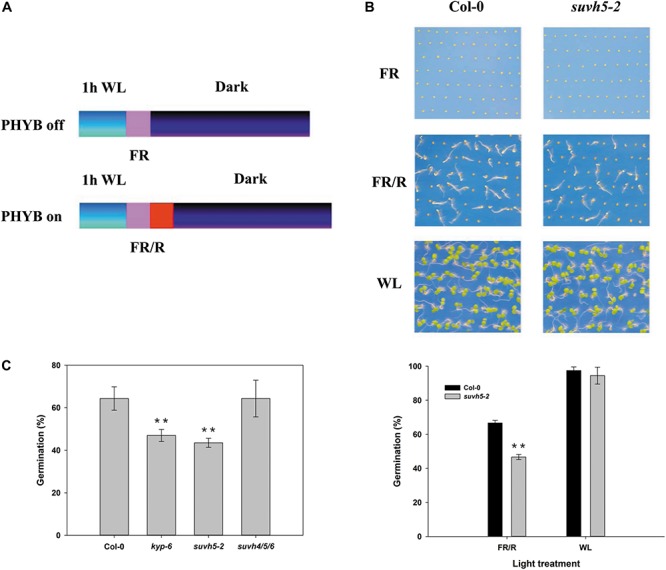
Analysis of the germination rate of *kyp-6*, *suvh5-2*, and *suvh4/5/6* mutant using PHYB-dependent seed germination assays. **(A)** Germination protocols of PHYB inactivated (PHYB-off, FR) and activated (PHYB-on, R) assays. **(B)** Germination patterns of Col-0 and *suvh5-2* under PHYB inactivated (FR), PHYB activated (R), and continuous white light (WL) conditions. Profiles (top) and germination rates (bottom) of Col-0 and *suvh5-2* on PHYB inactivated (FR), PHYB activated (R), and continuous white light (WL) conditions. After FR or R treatment, the seeds were kept in the dark for 48 h. Seeds were kept under continuous white light (WL) as a control. **(C)** Germination rates of Col-0, *kyp-6*, *suvh5-2*, and *suvh4/5/6* on PHYB activated (R) conditions. Germination frequencies were recorded at 48h after treatment. Values are shown as means ± SD (*n* = 3) (*t*-test, ^∗∗^*P* < 0.01, difference from Col-0).

To investigate whether histone methyltransferase SUVH5 plays a role in light-regulated seed germination, we examined the germination trait of a previously reported loss of function of *SUVH5* mutant *suvh5-2* (Ebbs and Bender, 2006) by PHYB-dependent germination protocol (Oh et al., 2004; Oh et al., 2006). 1 h after white light (WL) irradiation and surface sterilization, the seeds were exposed to 5 min far-red (FR) light (PHYB inactive, referred as FR or dark conditions) or followed by illumination with 5 min red light (PHYB activation, indicated as R or light conditions), and subsequently kept in the dark for 2 days (Figure 1A). Upon FR conditions, both wild-type Col-0 and *suvh5-2* seeds failed to germinate (the germination rates were 0) (Figure 1B). Next, we examined the germination rates of Col-0 and *suvh5-2* after R treatment. 66.6% of the wild-type seeds germinated 48 h after treatment, while *suvh5-2* seeds displayed relatively lower germination rates (46.6%) compared to the wild-type at the indicated time point (Figure 1B). As a control, seeds were kept under continuous white light (WL) and scored for germination (Figure 1B). Moreover, we also tested dynamics of germination rates of Col-0 and *suvh5-2* on PHYB activated (R) conditions after a long period. As shown in Supplementary Figure S1, except at 84 h, there were no significant germination rates difference in Col-0 and *suvh5-2* 48 h after treatment. The results indicated that SUVH5 may mainly modulate the germination kinetic in the initial phases and slightly affect the final rate of germination.

Previous study displayed that the plant SUPPRESSOR OF VARIEGATION 3-9 HOMOLOG (SUVH) family members, SUVH4, SUVH5, and SUVH6 act redundantly in regulating methylation of H3K9 and transposon silencing (Ebbs and Bender, 2006; Yu et al., 2017). We further examined the phenotype of the *suvh4* single mutant, *kyp-6* and the *suvh4/5/6* triple mutant (Ebbs and Bender, 2006). Similar to *suvh5-2*, *kyp-6* showed relatively lower germination rates compared to Col-0, which indicated that SUVH4 and SUVH5 may act redundantly in the control of seed germination. Surprisingly, *suvh4/5/6* triple mutant displayed a similar germination phenotype with wild-type (Figure 1C), which suggested that SUVH6 may act oppositely to SUVH4 and SUVH5 in regulating light-mediated seed germination. Together, these data indicated that SUVH5 may act as a positive regulator of PHYB-dependent seed germination.

### Expression Pattern of SUVH5 Is Regulated by Light in Imbibed Seeds

**FIGURE 2:**
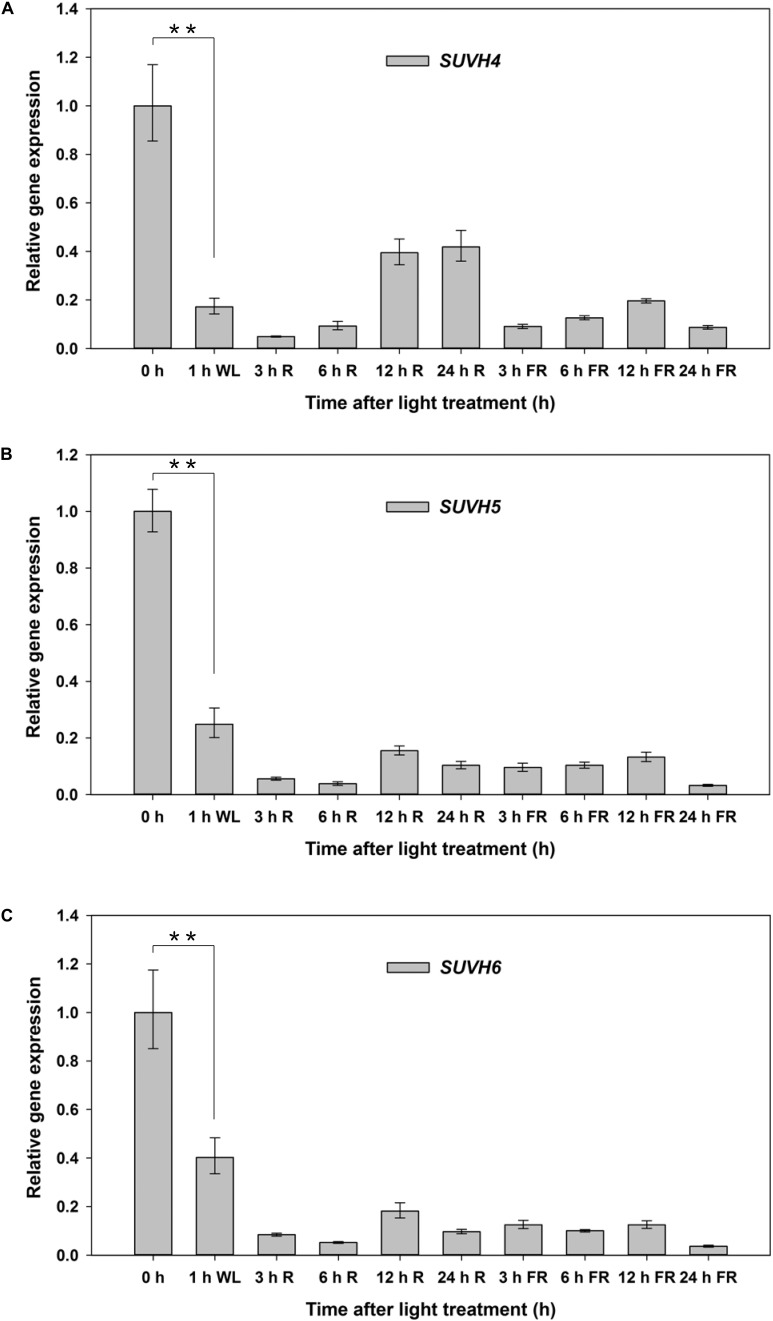
Expression patterns of *SUVH4/5/6* under R and FR conditions. Equal amount of Col-0 seeds were treated with FR or R light pulse and subsequently incubated in dark for indicated times before extracting mRNA. 0 h indicates dry seeds. *PP2A* was used as an internal control. Values are shown as means ± SD (*n* = 3) (*t*-test, ^∗∗^*P* < 0.01, difference from 0 h). **(A)** Expression patterns of *SUVH4* under R and FR conditions. **(B)** Expression patterns of *SUVH5* under R and FR conditions. **(C)** Expression patterns of *SUVH6* under R and FR conditions.

We further examined the expression patterns of *SUVH5* and its close homologs, *SUVH4* and *SUVH6* under FR and R conditions in germinating seeds. Seeds of Col-0 were treated with FR and FR/R exposure separately, after imbibed for indicated time (3, 6, 12, and 24 h), the seeds were harvested for gene expression analysis. *SUVH4*/5/*6* demonstrated a similar expression profile during light-mediated seed germination process (Figure 2). Relatively higher expression levels of *SUVH4/5/6* were detected in dry seeds (0 h as indicated). 1 h after imbibition, the transcripts of *SUVH4/5/6* were significantly decreased. Except for a recover of *SUVH4* expression 12 and 24 h after R treatment, both FR and R treatments decreased the expression of *SUVH4/5/6* in imbibed seeds compared to 0 h (Figure 2). Moreover, we also analyzed the expression of *SUVH4/5/6* in imbibed seeds under continuous FR and R irradiation. Except for continuous R light promoted *SUVH4* expression and a recover of *SUVH4/SUVH6* expression 12 h after continuous FR treatment, both continuous FR and R impulse repressed the expression of *SUVH4/5/6* in imbibed seeds compared to 0 h (Supplementary Figure S2). Collectively, these data suggested that the expression of *SUVH4/5/6* was regulated by light during the initial phase of seed germination.

### SUVH5 Regulates 24.6% of the Light-Responsive Transcriptome in Imbibed Seeds

To further study the function of SUVH5 in light-mediated seed germination, we examined the SUVH5-regulated transcriptome changes under R conditions by RNA-sequencing (RNA-seq) assays. Seeds of Col-0 and *suvh5-2* were treated under R conditions and kept in the dark, 24 h after imbibition, the seeds were harvested for RNA extraction, library construction, and high-throughput sequencing. To get reliable RNA-seq results, three independent biological replicated samples were harvest for analysis. Genes with more than 1.5-fold changes with statistical significance (adjusted *P* value < 0.05) were selected. Compared with the wild-type, 982 genes were up-regulated whereas only 101 genes were down-regulated in *suvh5-2* mutant, which suggested that SUVH5 may act mainly as a transcription repressor in light-mediated seed germination process (Supplementary Tables S1, S2).

**FIGURE 3:**
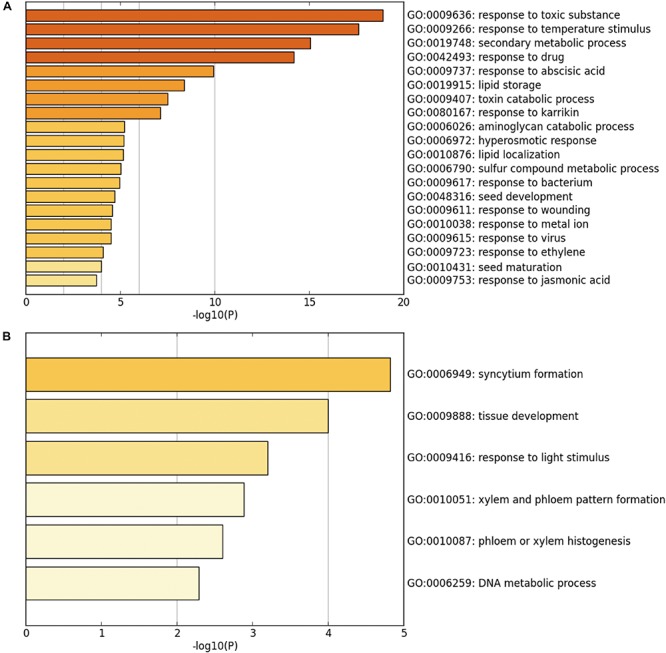
Chart of enriched ontology clusters of significantly expressed genes, which is repressed **(A)** or activated **(B)** by SUVH5 (*P* < 0.05).

Next, GO and functional clustering analysis of SUVH5-regulated genes were performed by Metascape software. We showed that the genes up-regulated in *suvh5-2* are mainly associated with the biological processes including response to toxic substance, response to temperature stimulus, secondary metabolic process, response to drug, response to abscisic acid, lipid storage, toxin catabolic processes, and response to karrikin (Figure 3A). In contrast, the genes down-regulated in *suvh5-2* are preferentially enriched in syncytium formation, tissue development, response to light stimulus, xylem and phloem pattern formation, phloem or xylem histogenesis, and DNA metabolic process (Figure 3B). Collectively, these results suggested that SUVH5 may integrate multiple internal and external factors to regulate many developmental processes, including light-regulated seed germination.

**FIGURE 4:**
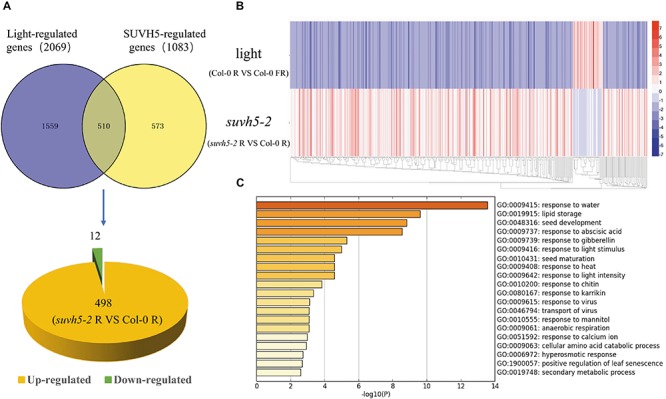
Genome-wide analysis of SUVH5-regulated transcriptome in light-mediated seed germination process. **(A)** Venn diagram shows the overlap of SUVH5- and light-regulated genes. Among these co-regulated genes, 498 were up-regulated whereas 12 were down-regulated in *suvh5*. **(B)** Heatmap of light and SUVH5 co-regulated genes. The bar indicates the fold change (*suvh5-2* R vs. Col-0 R and Col-0 R vs. Col-0 FR). **(C)** Chart of enriched ontology clusters of significantly expressed genes co-regulated by SUVH5 and light (*P* < 0.05).

Previous transcriptome analysis displayed that 2069 genes are regulated by light in imbibed seeds in *Arabidopsis* (Col-0 R vs. Col-0 FR) (Shi et al., 2013). Analysis in combination with SUVH5 and light-regulated transcriptomes demonstrated that the expression of about 24.6% (510) light-regulated genes was altered in *suvh5* mutant (Figure 4A and Supplementary Table S3). Interestingly, most of these genes (97.6%, 498) were down-regulated by light whereas up-regulated in *suvh5-2* (Figure 4A). Consistently, heatmap displayed that *suvh5-2* and light modulated the transcriptome changes in an opposite manner (Figure 4B). These data indicated that SUVH5 may act as a key positive regulator of light-regulated transcriptome in imbibed seeds.

Gene ontology analysis and functional clustering analysis revealed that SUVH5 and light co-regulated genes are mainly related to the processes including response to water, lipid storage, seed development, response to abscisic acid, response to gibberellin, response to light stimulus, seed maturation, response to heat, response to light intensity, response to chitin, and response to karrikin (Figure 4C). Collectively, these findings suggested that SUVH5 and light co-regulated many developmental processes by affecting the expression of the light-responsive genes related to multiple hormonal signaling pathways and development processes.

### SUVH5 Represses the Expression of Genes Related to ABA/GA Signaling Pathways and Regulates Endogenous ABA Contents in Imbibed Seeds

**FIGURE 5:**
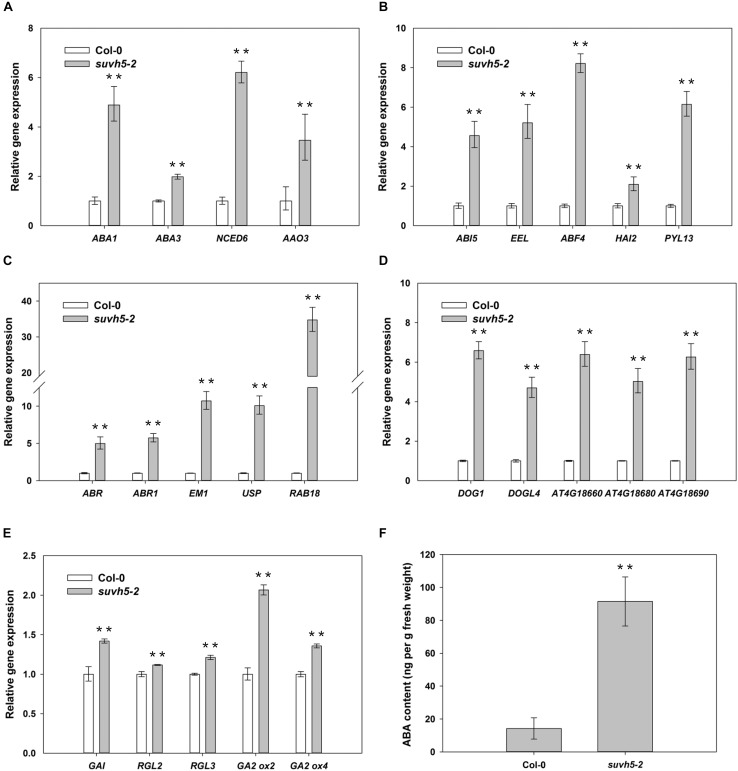
qRT-PCR analyses of the expression levels of ABA biosynthesis genes **(A)**, ABA signaling transduction genes **(B)**, ABA responsive genes **(C)**, *DOG* family genes **(D)**, GA signaling transduction/deactivating genes **(E)**, and analyses endogenous abscisic acid (ABA) contents **(F)** in imbibed Col-0 and *suvh5-2* seeds under R conditions. Equal amount of Col-0 and *suvh5-2* mutant seeds were treated with R light pulse and subsequently incubated in dark for 24 h before extracting mRNA and ABA. *PP2A* was used as an internal control of qRT-PCR analyses. Values are shown as means ± SD (*n* = 3) (*t*-test, ^∗∗^*P* < 0.01, difference from Col-0).

It’s well known that ABA plays a predominant negative role in the regulation of seed germination. Our RNA-seq analysis demonstrated that the expression of a large subset of ABA signaling-related genes, including ABA biosynthesis genes (*ABA1*, *ABA3*, *NCED6*, and *AAO3*), ABA signal transduction genes (*ABI5*, *EEL*, *ABF4*, *HAI2*, and *PYL13*) and ABA-responsive genes (*ABR*, *ABR1*, *EM1*, *USP*, *RAB18*, and et al.) were up-regulated in *suvh5* mutant compared with wild-type upon R conditions (Supplementary Table S1, S5). Further quantitative RT-PCR (qRT-PCR) analysis displayed that relatively higher expression levels of these genes were detected in imbibed *suvh5-2* seeds compared with wild-type (Figures 5A–C). Consistently, we showed that the ABA content in *suvh5-2* seeds was 6.5-fold higher than that in wild-type (Figure 5F). GA is another critical plant hormone in the regulation of seed germination, we further analyzed the expression of genes relate to GA signaling and metabolism processes. DELLA (GAI, RGA, RGL1, RGL2, and RGL3) proteins are master negative components in GA signaling, and RGL2 is the main repressor of seed germination (Nelson and Steber, 2016). GA2ox is crucial catabolic enzyme of bioactive GAs and negatively regulated GA metabolism (Liu and Hou, 2018). Increased expression level of *GAI*, *RGL2*, *RGL3*, *GA2ox2*, and *GA2ox4* was examined in imbibed *suvh5* seeds compared with wild-type under R condition (Figure 5E), which suggested that SUVH5 may also regulate GA signaling and GA catabolism in the control of seed germination. Together, these data suggested that SUVH5 may promote light-mediated seed germination by modulating the balance of ABA and GA in imbibed seeds.

### SUVH5 Represses the Expression of a Family of DOG Genes in Imbibed Seeds

*DELAY OF GERMINATION 1* is a master regulator of seed dormancy and belongs to a plant-specific gene family with other four *DOG1*-like genes, *At4g18660*, *At4g18680*, *At4g18690*, and *DOGL4* (Bentsink et al., 2006). RNA-seq analysis revealed that the expression of some *DOG1-*like genes was up-regulated in *suvh5-2* mutant (Supplementary Table S1). We further examined the transcripts of *DOG1* and *DOG1-*like genes by qRT-PCR assays. The levels of the expression of *DOG1*, *At4g18660*, *At4g18680*, *At4g18690*, and *DOGL4* were significantly up-regulated in *suvh5-2* compared with wild-type seeds under R conditions (Figure 5D), which indicated that SUVH5 may increase seed germination by repressing the expression of *the DOG* genes.

### SUVH5 Represses ABA Signaling and DOG Genes by Histone H3K9 Dimethylation

**FIGURE 6:**
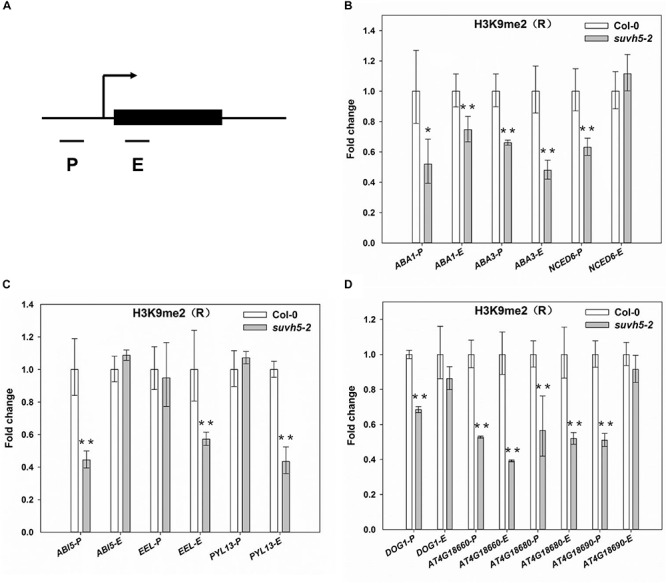
ChIP-qPCR analyses of H3K9me2 levels of SUVH5-regulated genes. **(A)** Schematic diagram of the regions for ChIP analysis. P and E indicate proximal promoter and first exon regions, respectively. ChIP-qPCR analyses of the histone H3K9me2 levels at the promoter and first exon regions of ABA biosynthesis genes **(B)**, ABA signaling transduction genes **(C)**, and *DOG* family genes **(D)** in imbibed Col-0 and *suvh5-2* seeds under R conditions. Equal amount of Col-0 and *suvh5-2* mutant seeds were treated with R light pulse and subsequently incubated in dark for 24 h before ChIP-qPCR analyses. The amounts of DNA after ChIP were quantified and normalized to *TA3*, the relative enrichment refers to the H3K9me2 enrichment vs. the histone H3 occupancy. Values are shown as means ± SD (n = 3) (*t*-test, ^*^P < 0.05, ^∗∗^*P* < 0.01, difference from Col-0).

Previous studies suggested that SUVH5 modulates transcriptional gene silencing through histone H3K9 methyltransferase activity (Ebbs and Bender, 2006; Rajakumara et al., 2011; Yu et al., 2017), and the SRA domain of SUVH5 is required for the accumulation of the H3K9 dimethylation (Rajakumara et al., 2011; Yu et al., 2017). We then analyzed the levels of histone H3K9me2 of the ABA signaling and *DOG* genes in wild-type and *suvh5*-2 upon R conditions by chromatin immunoprecipitation in combination with quantitative PCR (ChIP-qPCR) assays. Since previously genome-wide profiling indicated that H3K9me2 modification occurred in target gene promoters or in gene bodies (Zhou et al., 2010), then the regions proximal to the transcriptional starting sites (P), and the first exon regions (E) of these genes were selected for analysis (Figure 6A).

For the ABA biosynthesis genes, the levels of H3K9me2 were significantly decreased at the promoter and first exon regions of *ABA1* and *ABA3* as well as the promoter region of *NCED6* in imbibed *suvh5* seeds compared with wild-type (Figure 6B). For the ABA signal transduction-related genes, a significant decrease of H3K9me2 level was detected at the promoter of *ABI5* and the exon regions of *EEL* and *PYL13* in *suvh5* mutant (Figure 6C). Furthermore, relatively lower levels of H3K9me2 were detected at the promoters of *DOG1*, *At4g18660*, *At4g18680*, and *At4g18690*, as well as at the exons of *At4g18660* and *At4g18680* in *suvh5* mutant compared with wild-type (Figure 6D). Together, these data suggested that SUVH5 represses the expression of ABA signaling and *DOG* genes by histone H3K9 dimethylation.

## Discussion

In this work, we present evidence indicating that SUVH5 is a positive component of light-mediated seed germination process. Loss of function of *SUVH5* results in decreased germination and leads to 24.6% of light-responsive transcriptome changes. Moreover, SUVH5 represses the expression of ABA signaling and *DOG* genes via dimethylation of histone H3 at lysine 9.

Abscisic acid is a critical plant hormone in regulating seed germination and dormancy. Previous studies demonstrated that loss of function of ABA biosynthesis mutant *aba1* displayed reduced seed dormancy and faster germination rate both in *Arabidopsis* and tobacco (Koornneef, 1982; Grappin et al., 2000). Mutation of *aba3* impaired in ABA biosynthesis and resulted in reduced seed dormancy (Léon-Kloosterziel et al., 1996). *NCED6*, another key regulator of ABA biosynthesis, also functions in the induction of seed dormancy (Lefebvre et al., 2006). In present work, we showed that SUVH5 represses the expression of these ABA biosynthesis genes by dimethylation of histone H3K9, which indicated that SUVH5 may promote light-mediated seed germination by decreasing ABA content in imbibed seeds. Furthermore, bZIP-type transcription factor *ABI5* maintains seed dormancy by activating the expression of genes including seed storage protein genes (Piskurewicz et al., 2008; Kanai et al., 2010; Wang et al., 2011). *EEL*, a transcription factor homologous to *ABI5*, is also able to bind to the ABA-responsive elements (ABRE) of seed storage protein genes during late embryogenesis (Bensmihen et al., 2005). Repression of *ABI5* and *EEL* transcripts by SUVH5 indicated that SUVH5 may act to accelerate the launch of germination in imbibed seeds. Moreover, PYR/PYL/RCAR proteins are intracellular ABA receptors regulating ABA-dependent gene expression. Recent works demonstrated that *pyl* duodecuple mutant, *pyr1pyl1/2/3/4/5/7/8/9/10/11/12* is extremely insensitive to ABA effects on seed germination, whereas transgenic plants overexpressing *PYL13* show increased ABA sensitivity in seed germination (Zhao et al., 2013; Fuchs et al., 2014). Repressing of *PYL13* expression by SUVH5 indicated that SUVH5 may decrease ABA perception thus restrain ABA signal transduction in imbibed seeds. In addition, a number of ABA-responsive genes, such as *ABR*, *ABR1*, *EM1*, *USP*, and *RAB18* were up-regulated in *suvh5* mutant, which confirmed a negative role of SUVH5 in ABA signal transduction in imbibed seeds. Together, these findings revealed that SUVH5 may promote seed germination via inhibiting both ABA biosynthesis and ABA signal transduction pathways in imbibed seeds.

Diverse epigenetic modifications, such as DNA methylation, histone modification and chromatin-remodeling, have been reported to play critical roles in regulating seed germination (Dean Rider et al., 2003; Perruc et al., 2007; Saez et al., 2008; Cho et al., 2012; Luo et al., 2012; Zhou et al., 2013; Liu et al., 2014; Gu et al., 2017; Kawakatsu et al., 2017). Recent work displayed that the expression of *DOGL4*, a paralogous gene of *DOG1*, is regulated by DNA demethylase ROS1-mediated DNA demethylation (Zhu et al., 2007, 2018). In present work, we showed that the expression of *DOGL4* is also repressed by SUVH5-mediated histone H3K9 dimethylation. Interestingly, structure-based studies indicated that a functional SUVH5 SRA domain is required for both DNA methylation and accumulation of the H3K9me2 (Rajakumara et al., 2011). These findings suggested that SUVH5 may repress the expression of *DOG* genes through both histone H3K9 dimethylation and DNA methylation manners. It will be meaningful to study interplay of SUVH5 and ROS1 in the regulation of *DOG* genes expression in light-mediated process. Moreover, it’s well known that the epigetic factors usually act in multi-protein complexes in regulating gene expression (Liu et al., 2014). Previous studies demonstrated that RPD3-HDA1-type histone deacetylase HDA6 represses the expression of the ABA pathway genes by regulating the levels of H3ac, H3K4me3 and H3K9me2 (Chen et al., 2010; Chen and Wu, 2010; Luo et al., 2012). In present work, we also showed that SUVH5 represses the expression of ABA signaling-related genes via H3K9me2. A recent study reported that SUVH4/5/6 and HDA6 act in a same protein complex (Yu et al., 2017). These findings strongly suggested that SUVH5 may associate with HDA6 in the regulation of light-mediated seed germination. Further analysis of SUVH5-containing protein complexes will help to elucidate its role of in light-regulated seed germination process.

In imbibed seeds, *SUVH5* and *SUVH6* displayed similar expression patterns after R and FR irradiation, which indicated that their transcripts might be regulated by the same upstream light-responsive transcription regulators. However, the similar gene expression profiles do not mean they play same roles in light-mediated seed germination. Phenotypic analysis of *suvh4*, *suvht5* and *suvh4/5/6* triple mutant suggested that SUVH6 may act oppositely to SUVH4 and SUVH5 and negatively regulate light-mediated seed germination. SUVH proteins generally functions as repressors of gene expression via histone H3K9 dimethylation (Ebbs and Bender, 2006). A recent study demonstrated that KPY/SUVH5/SUVH6 proteins have distinct methylated DNA binding preference, which suggested that these proteins may target different downstream genes (Li et al., 2018). In present work, we showed that SUVH5 represses the expression of ABA biosynthesis, ABA signal transduction as well as *DOG* genes via histone H3K9 dimethylation. SUVH6 may repress light-mediated seed germination by repressing the expression of some other target genes, such as GA biosynthesis and GA signal transduction related genes in imbibed seeds. Further genome-wide analysis of the downstream genes of SUVH6 will help to explore its role in light-mediated seed germination.

In summary, we identified histone methyltransferase SUVH5 as a positive regulator in light-mediated seed germination. Upon R or strong light conditions, SUVH5 depresses the expression of ABA signaling and *DOG* genes via dimethylation of H3K9, resulting in reduced levels of ABA contents and increased germination kinetic in imbibed seeds. Moreover, SUVH5 may increase GA levels in imbibed seeds by repress the expression of GA catabolic genes. Ultimately, the changed balance between ABA and GA by SUVH5 leads to promotion of seed germination.

## Data Availability

All datasets generated for this study are included in the manuscript and/or the Supplementary Files.

## Author Contributions

XL designed the study. DG conducted most of the experiments. RJ, CH, and TP conducted some of the experiments. MZ, JD, and CX contributed with some materials and reagents. DG and XL analyzed the data and wrote the manuscript.

## Conflict of Interest Statement

The authors declare that the research was conducted in the absence of any commercial or financial relationships that could be construed as a potential conflict of interest.

## Footnotes

1 http://www.arabidopsis.org/

2 http://cole-trapnell-lab.github.io/cufflinks/

3 http://metascape.org

## Supplementary Material

FIGURE S1 Dynamics of germination rates of Col-0 and *suvh5-2* on PHYB activated (R) conditions. Germination frequencies were recorded at indicated time points after treatment. Values are shown as means ± SD (*n* = 3) (*t*-test, ^*^*P* < 0.05, ^∗∗^*P* < 0.01, difference from Col-0).

FIGURE S2 Expression patterns of *SUVH4/5/6* under continuous R light and FR light conditions. Equal amount of Col-0 seeds were treated with continuous FR or R light pulse at 22°C for indicated times before extracting mRNA. 0 h indicates dry seeds. *PP2A* was used as an internal control. Values are shown as means ± SD (*n* = 3). **(A)** Expression patterns of *SUVH4* under continuous R and FR conditions. **(B)** Expression patterns of *SUVH5* under continuous R and FR conditions. **(C)** Expression patterns of *SUVH6* under continuous R and FR conditions.

TABLE S1 Genes up-regulated in *suvh5-2* in R conditions.

TABLE S2 Genes down-regulated in *suvh5-2* in R conditions.

TABLE S3 SUVH5 and light co-regulated genes.

TABLE S4 Primer pairs used in this study.

TABLE S5 List of ABA signaling-related genes repressed by SUVH5 in imbibed seeds.
