# Supplementary figures and images for: Arabidopsis Histone Methyltransferase SUVH5 Is a Positive Regulator of Light-Mediated Seed Germination
Source: Front Plant Sci. 2019 Jun 27;10:841. doi: 10.3389/fpls.2019.00841 (PMC6610342; doi:10.3389/fpls.2019.00841)

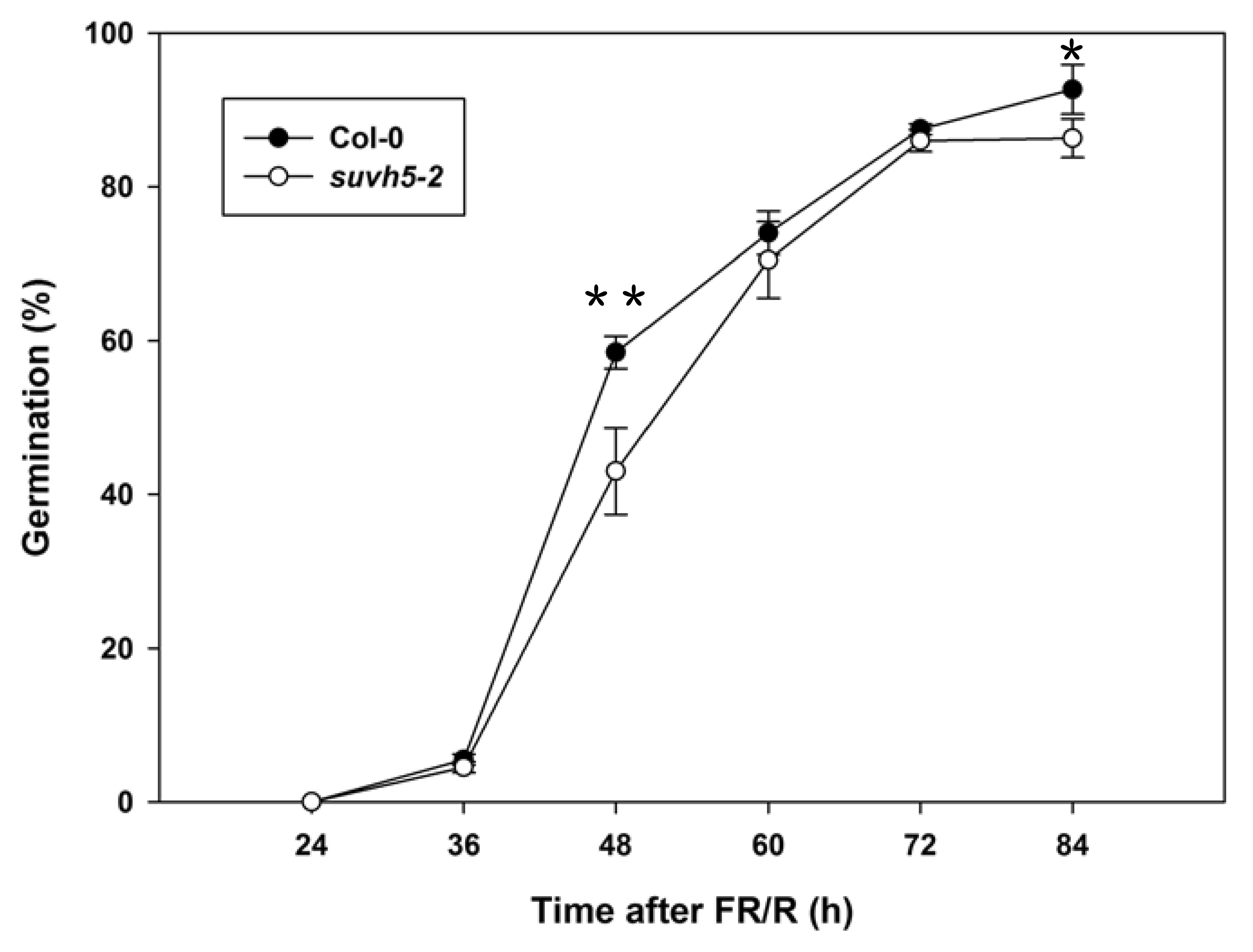

Supplement: FIGURE S1 — Dynamics of germination rates of Col-0 and suvh5-2 on PHYB activated (R) conditions. Germination frequencies were recorded at indicated time points after treatment. Values are shown as means ± SD (n = 3) (t-test, *P < 0.05, ∗∗P < 0.01, difference from Col-0). [file Image_1.TIF]

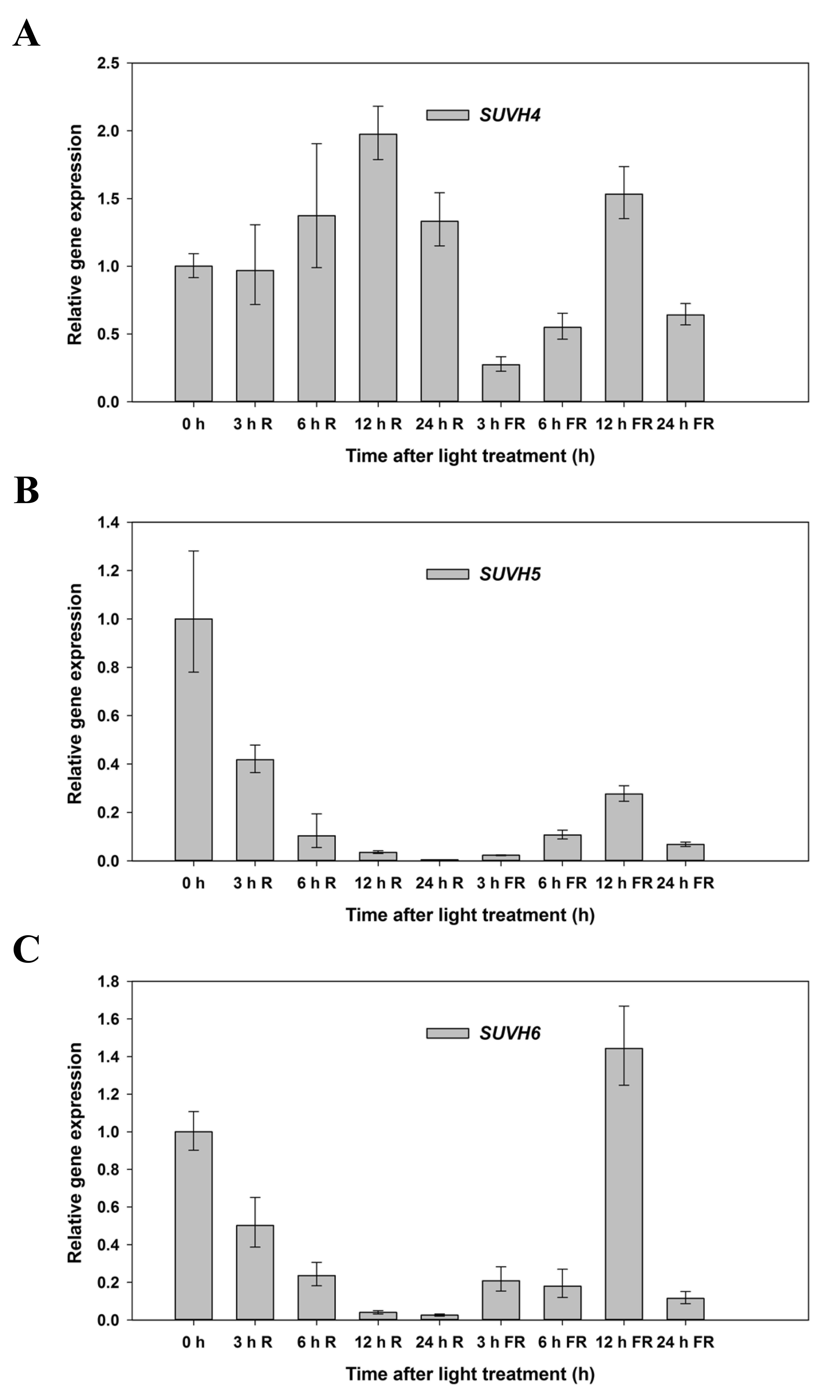

Supplement: FIGURE S2 — Expression patterns of SUVH4/5/6 under continuous R light and FR light conditions. Equal amount of Col-0 seeds were treated with continuous FR or R light pulse at 22°C for indicated times before extracting mRNA. 0 h indicates dry seeds. PP2A was used as an internal control. Values are shown as means ± SD (n = 3). (A) Expression patterns of SUVH4 under continuous R and FR conditions. (B) Expression patterns of SUVH5 under continuous R and FR conditions. (C) Expression patterns of SUVH6 under continuous R and FR conditions. [file Image_2.TIF]
