# Supplementary material for: Arabidopsis Histone Methyltransferase SUVH5 Is a Positive Regulator of Light-Mediated Seed Germination
Source: Front Plant Sci. 2019 Jun 27;10:841. doi: 10.3389/fpls.2019.00841 (PMC6610342; doi:10.3389/fpls.2019.00841)
Supplement: TABLE S4 — Primer pairs used in this study. [file Table_4.DOCX]

Table S4. Primers pairs used in this work

| Primer | Sequence (5’-3’) |
| --- | --- |
| Primer pairs for qRT-PCR | |
| PP2A RT pr1 | GTGACTTGGTTGAGCATTTCACTCC |
| PP2A RT pr2 | GAGCTGATTCAATTGTAGCAGCAAACT |
| SUVH4 RT pr1 | TTCGCAGCTGACAACATTTC |
| SUVH4 RT pr2 | AACAACAATGCCGAAAAAGG |
| SUVH5 RT pr1 | TTGCAGGAAAAGCATCACTG |
| SUVH5 RT pr2 | CTAGGAAGCCCACCATTTGA |
| SUVH6 RT pr1 | TGCCTCTGAAAACGTGAGTG |
| SUVH6 RT pr2 | CTAGCGGCTGAGTTTGATCC |
| ABA1 RT pr1 | GATGCAGCCAAATATGGGTCAAGG |
| ABA1 RT pr2 | GCCATTGCATGGATAATAGCGACTC |
| ABA3 RT pr1 | CAAGCTTGTGTGTATTATCGTCT |
| ABA3 RT pr2 | TATACAGGTCCAGTAACAGATTTG |
| NCED6 RT pr1 | ACCGGGTCGGATATAAATTGGGTTG |
| NCED6 RT pr2 | CCCGGGTTGGTTCTCCTGATTC |
| AAO3 RT pr1 | TCGATGTGCAGGTCAAAGAATTGC |
| AAO3 RT pr2 | AACATCGGATGAACCTCGAAAAAGC |
| ABI5 RT pr1 | CAATAAGAGAGGGATAGCGAACGAG |
| ABI5 RT pr2 | CGTCCATTGCTGTCTCCTCCA |
| EEL RT pr1 | GGAGGCTAAAGGAGGTGGAGAAGA |
| EEL RT pr2 | AGAGAAGCAGAGTTTGTTCGCCG |
| ABF4 RT pr1 | TGAGCTGAAAGAAACGTCGAAGC |
| ABF4 RT pr2 | TCCGGTTAATGTCCTTCTCAAGCA |
| HAI2 RT pr1 | TTGGGGTGAAACTGTGATGA |
| HAI2 RT pr2 | CGGACGATCAGGCTTATGAT |
| PYL13 RT pr1 | TTTCGTCAAAAGTTGCACCA |
| PYL13 RT pr2 | GCCTATGGTTACCGCCAATA |
| ABR RT pr1 | CCACTGGCCAGACTAAGGAG |
| ABR RT pr2 | CTTTTGTTCATGCCGGTCTT |
| ABR1 RT pr1 | AAAGGAGGAGAGAGGTGGAGGA |
| ABR1 RT pr2 | ACTTTAGAAGTAGAGCTACCACCG |
| EM1 RT pr1 | TTGCTGAAGGAAGGAGCAAG |
| EM1 RT pr2 | CGCTCTCCACCAGATTTTTC |
| USP RT pr1 | GTTGCGATTGATGAGAGCAA |
| USP RT pr2 | GAAGCAGTTCCACCAGAAGG |
| RAB18 RT pr1 | CAGCTCTAGCTCGGAGGATG |
| RAB18 RT pr2 | TCTTGTCCATCATCCCCTTC |
| DOG1 RT pr1 | ACACAAACACGCAAACCAAA |
| DOG1 RT pr2 | CTCAGGGATGCGTTGAGATT |
| DOGL4 RT pr1 | CTCTTGGTTAACCGGATGGA |
| DOGL4 RT pr2 | ACCGCTCCATCTCTCTCTCA |
| AT4G18660 RT pr1 | TAATCGGACAAGCTGACGTG |
| AT4G18660 RT pr2 | GCATCGCCAAATGAAGTTTT |
| AT4G18680 RT pr1 | CCGTCGTGGAACACTCCTAT |
| AT4G18680 RT pr2 | AAGCTGTGTCGCGGTAAGAT |
| AT4G18690 RT pr1 | GTGGTGGTGGAAGATGCTTT |
| AT4G18690 RT pr2 | CCCACTCGTGCAATGATATG |
| GAI RT pr1 | AGCGTCATGAAACGTTGAGTCAGTG |
| GAI RT pr2 | TGCCAACCCAACATGAGACAGC |
| RGL2 RT pr1 | CCGACCCGAATCTGAAACCTTAGTG |
| RGL2 RT pr2 | AAGCGCTTCGTTGAACCTATCGAG |
| RGL3 RT pr1 | CAAACGAAACCTCTAATCGCTGCAT |
| RGL3 RT pr2 | GGGCGAAATTGTCACAAAACGAAAC |
| GA2ox2 RT pr1 | CTCTTCTCAATCATAAAACCCTT |
| GA2ox2 RT pr2 | CAAAACACTATCCATTTCCTTCT |
| GA2ox4 RT pr1 | TAGAGGCGAAAATATCACCGTT |
| GA2ox4 RT pr2 | AGACGAGGGACATCTAAACGG |
| Primer pairs used for ChIP-qPCR analysis | |
| TA3-F | GATTCTTACTGTAAAGAACATGGCATTGAGAGA |
| TA3-R | TCCAAATTTCCTGAGGTGCTTGTAACC |
| ABA1-P pr1 | GCTGATCACGTCACGTTCAA |
| ABA1-P pr2 | CAAATGATGGAGGAGGAGGA |
| ABA1-E pr1 | CGGCGTTAGTTGAGAAGGAG |
| ABA1-E pr2 | ATCGGGCCTCTGTATTTTCC |
| ABA3-P pr1 | AAATAAACTCGAACCCGAACC |
| ABA3-P pr2 | GTCCTAAGCGTTTGGGTATTC |
| ABA3-E pr1 | CGGGAACGACCATCTTTCT |
| ABA3-E pr2 | GTCGCGGATCTCTTGAATGT |
| NCED6-P pr1 | GATTCGACACCTATTTAGGTTC |
| NCED6-P pr2 | ATGTTCTCTACTATAATGTGGAGT |
| NCED6-E pr1 | GAAGTGGTTGGTCAGATTCC |
| NCED6-E pr2 | TACCGGCAGCTGTAACTAAC |
| ABI5-P pr1 | AGCATTCCATTGGTCGAAAG |
| ABI5-P pr2 | GCCGGAGAATTTTGACTGAA |
| ABI5-E pr1 | TTGACGTCAGAGCGAGAAGT |
| ABI5-E pr2 | CCAAAGTTCTTGCCGTTCTC |
| EEL-P pr1 | GGCACAGTTTAAAGTTCGAGTG |
| EEL-P pr2 | CCAAACATACATGCTCTCTTCTC |
| EEL-E pr1 | CTCAGTCATTAACGAGGCAGAA |
| EEL-E pr2 | CGAGGTAACGTCAAGCTTCC |
| PYL13-P pr1 | TCAGAATACCCTTCACAGATGG |
| PYL13-P pr2 | TGTACACAAGTATTGACTGCCTTG |
| PYL13-E pr1 | TTTCGTCAAAAGTTGCACCA |
| PYL13-E pr2 | GCCTATGGTTACCGCCAATA |
| DOG1-P pr1 | TTTGGAACAACAACTCGCACT |
| DOG1-P pr2 | GGACAGAAAATGTTTACTTGGACAC |
| DOG1-E pr1 | ACACAAACACGCAAACCAAA |
| DOG1-E pr2 | CTCAGGGATGCGTTGAGATT |
| AT4G18660-P pr1 | GCTGCGAAAATAGACCATCTG |
| AT4G18660-P pr2 | CTTAAACCTACGGAGGACCAT |
| AT4G18660-E pr1 | ATCGGAGGAGGAGAGTCGAT |
| AT4G18660-E pr2 | CACGTCAGCTTGTCCGATTA |
| AT4G18680-P pr1 | ACCCACCGCGTAGTGATTAG |
| AT4G18680-P pr2 | TTCAACCCCATAACTCGAAGA |
| AT4G18680-E pr1 | GATGATCTCAAAGAAGCACTCA |
| AT4G18680-E pr2 | ACGACGGTGCAAAGTAGTGA |
| AT4G18690-P pr1 | TGTTTCCGGCTAAAGCAAAG |
| AT4G18690-P pr2 | CATTGGATTGGTGCATGTATGT |
| AT4G18690-E pr1 | GTGGATGAGTGTGCAGGCTA |
| AT4G18690-E pr2 | GCGAGAGAGCTCAGATCGTT |
