# Supplementary material for: Arabidopsis Histone Methyltransferase SUVH5 Is a Positive Regulator of Light-Mediated Seed Germination
Source: Front Plant Sci. 2019 Jun 27;10:841. doi: 10.3389/fpls.2019.00841 (PMC6610342; doi:10.3389/fpls.2019.00841)
Supplement: TABLE S5 — List of ABA signaling-related genes repressed by SUVH5 in imbibed seeds. [file Table_5.DOCX]

Table S5. List of genes related to ABA signaling pathway which repressed by SUVH5 in imbibed seeds.

| Gene name | Description | Classification |  |
| --- | --- | --- | --- |
| ABA1 | zeaxanthin epoxidase (ZEP) | ABA biosynthesis |  |
| ABA3 | abscisic aldehyde oxidase 3 | ABA biosynthesis |  |
| NCED6 | nine-cis-epoxycarotenoid dioxygenase 6 | ABA biosynthesis |  |
| AAO3 | molybdenum cofactor sulfurase | ABA biosynthesis |  |
| ABI5 | Basic-leucine zipper transcription factor family protein | ABA signal transduction |  |
| EEL | Basic-leucine zipper transcription factor family protein | ABA signal transduction |  |
| ABF4 | ABRE binding factor 4 | ABA signal transduction |  |
| HAI2 | highly ABA-induced PP2C protein 2 | ABA signal transduction |  |
| PYL13 | PYR1-like 13 | ABA signal transduction |  |
| ABR | Late embryogenesis abundant protein (LEA) family protein | ABA response |  |
| ABR1 | Integrase-type DNA-binding superfamily protein | ABA response |  |
| EM1 | Stress induced protein | ABA response |  |
| USP | Adenine nucleotide alpha hydrolases-like superfamily protein | ABA response |  |
| RAB18 | ras-related small GTPase | ABA response |  |
|  |  |  |  |
|  |  |  |  |
|  |  |  |  |
|  |  |  |  |
